# A systematic pan-cancer analysis of the gasdermin (GSDM) family of genes and their correlation with prognosis, the tumor microenvironment, and drug sensitivity

**DOI:** 10.3389/fgene.2022.926796

**Published:** 2022-08-08

**Authors:** Yufu Zheng, Didi Yuan, Fei Zhang, Renkuan Tang

**Affiliations:** Department of Forensic Medicine, College of Basic Medicine, Chongqing Medical University, Chongqing, China

**Keywords:** pyroptosis, GSDM family, tumor microenvironment, drug sensitivity, prognosis

## Abstract

**Background:** Pyroptosis is a programmed cell death process mediated by the gasdermin (GSDM) protein. However, limited research has been conducted to comprehensively analyze the contribution of the GSDM family in a pan-cancer setting.

**Methods:** We systematically evaluated the gene expression, genetic variations, and prognostic values of the GSDM family members. Furthermore, we investigated the association between the expression of GSDM genes and immune subtypes, the tumor microenvironment (TME), the stemness index, and cancer drug sensitivities by means of a pan-cancer analysis.

**Results:** GSDM genes were highly upregulated in most of the tested cancers. Low-level mutation frequencies within GSDM genes were common across the examined types of cancer, and their expression levels were associated with prognosis, clinical characteristics, TME features, and stemness scores in several cancer types, particularly those of the urinary system. Importantly, we found that the expressions of *GSDMB*, *GSDMC*, and *GSDMD* were higher in kidney carcinomas, and specifically kidney renal clear cell carcinoma (KIRC); which adversely impacted the patient outcome. We showed that *GSDMD* was potentially the most useful biomarker for KIRC. The drug sensitivity analysis demonstrated that the expressions of GSDM genes were correlated with the sensitivity of tumor cells to treatment with chemotherapy drugs nelarabine, fluphenazine, dexrazoxane, bortezomib, midostaurin, and vincristine.

**Conclusion:** GSDM genes were associated with tumor behaviors and may participate in carcinogenesis. The results of this study may therefore provide new directions for further investigating the role of GSDM genes as therapeutic targets in a pan-cancer setting.

## Introduction

Pyroptosis is a type of programmed cell death primarily mediated by the family of pore-forming protein gasdermins (GSDMs) (Shi et al., 2017). When cleaved by caspase proteins, the GSDM family participates in pyroptotic pore formation in the cell membrane. These cause extensive water influx from the extracellular space, leading to cell swelling and rupture (Tsuchiya, 2021). Strong evidence has demonstrated that the pyroptosis process is highly involved in the pathogenesis and progression of cancer by inducing GSDMD-dependent caspase-1 inflammasome pathways and other GSDM-dependent non-inflammasome signaling cascades (Johnson et al., 2018; Pizato et al., 2018; Wang et al., 2019; Yu et al., 2019). Therefore, investigations of GSDM genes may provide a more comprehensive overview of carcinogenesis and shed light on novel therapeutic strategies.

The majority of GSDM family members share an N-terminal pore-forming effector domain and a C-terminal repressor domain (Ding et al., 2016; Kuang et al., 2017). Six types of GSDMs paralogous genes, namely *GSDMA*, *GSDMB*, *GSDMC*, *GSDMD*, and *GSDME* [also termed deafness, autosomal dominant 53 (*DFNA5*)], and pejvakin [*PJVK*; also termed deafness, autosomal recessive 59 9*DFNB59*)], have been found in humans (Liu et al., 2021). Specifically, *GSDMA* is primarily localized to gastric and skin epithelia; *GSDMB* is found in the esophagus, liver, and colon; *GSDMC* is located in keratinocytes, the trachea, and the spleen; *GSDMD* is widely distributed in various immune cells, the placenta, the esophagus, and the gastrointestinal tract epithelium; *GSDME* is primarily found in the cochlea and the placenta; while, *PJVK* is found in the heart and liver. Recent studies have revealed that the GSDM family of genes plays a dual role in tumor pathogenesis and progression (Xia et al., 2019). It has been reported that while *GSDMA* is silenced in gastric cancer, GSDMB is overexpressed in several types of cancers (e.g., breast and gastric cancers) (Hergueta-Redondo et al., 2016). Moreover, studies have shown that the expression levels of GSDMC and GSDME proteins were significantly upregulated in colorectal cancer tissues. *GSDMC* is regulated by transforming growth factor-beta (TGF-β) signaling and is associated with tumor cell proliferation (Miguchi et al., 2016). *GSDME* participates in tumorigenesis and colorectal tumor cell proliferation, specifically through its association with the extracellular signal-regulated kinase 1/2 (ERK1/2) pathway. *GSDME* has also been shown to exert potentially tumor-suppressive effects in models of intestinal cancer (Croes et al., 2019; Tan et al., 2020). GSDMD was found to be highly downregulated in human colorectal tumor samples and serves as an important prognostic molecule in tumor therapy (Wu et al., 2020). However, studies investigating the role of GSDM genes in various tumors are limited, and further comprehensive analyses are warranted to clarify their molecular characteristics in a pan-cancer setting.

In the present study, we used data from The Cancer Genome Atlas (TCGA) project and the cBioPortal database, to comprehensively analyze the landscape of the GSDM gene expression status and evaluate the genetic alteration of this set of genes in various cancer types. In addition, we determined the prognostic value of the GSDM gene family by examining clinical traits in a pan-cancer setting using the Kaplan–Meier plotter and data from the University of California Santa Cruz (UCSC) database. Subsequently, we investigated the potential correlation between the expression of GSDM genes and tumor microenvironment (TME) characteristics, as well as the stemness index. An investigation of the association between GSDM genes and numerous dynamic immune modulators, tumor mutational burden (TMB), and microsatellite instability (MSI) was also conducted. Finally, correlations between GSDM gene expression and cancer drug sensitivity were also explored in various aspects of cancer using public resources.

## Materials and methods

### Data resources

All pan-cancer data were obtained from the UCSC Xena project (https://xena.ucsc.edu/), which contains 11,768 samples and 33 tumor types originating from the TCGA database. We extracted the mRNA data of six pyroptosis-associated genes provided by the TCGA Pan-Cancer Atlas. The pan-cancer immune subtype data were also retrieved from the UCSC database; they included six immune groups: Immune C1 (Wound Healing); Immune C2 [interferon gamma (IFN-γ) Dominant]; Immune C3 (Inflammatory); Immune C4 (Lymphocyte Depleted); Immune C5 (Immunologically Quiet); and Immune C6 (TGF-β Dominant). Survival data in relation to the expression of GSDM genes in the pan-cancer setting were obtained from TCGA and Kaplan–Meier Plotter databases. Data on the DNA methylation-based stemness score and RNA-based stemness score were also downloaded from the UCSC Pan-Cancer project. The MSI data for each cancer type were obtained from a published study (Bonneville et al., 2017). Furthermore, we obtained the drug sensitivity data from the CellMiner™ database to analyze the correlation between GSDM gene expression and drug sensitivity.

### Data processing and statistical analysis

After merging the GSDM gene expression data to create the pan-cancer dataset, batch effects between different cancers were corrected using the “removeBatchEffect” function of the “limma” package (https://doi.org/doi:10.18129/B9.bioc.limma) of R software, using empirical Bayes algorithms. We used the Wilcoxon rank-sum test to investigate the differences in the expression of GSDM genes between malignant tissues and matched normal samples for each cancer type. Genomic alterations were evaluated using the cBioPortal website. The Kruskal–Wallis test was also used to compare the differences in gene expression patterns among the six immune subtypes (C1–C6). Next, we then analyzed the expression patterns of GSDM genes and investigated their correlation with different disease stages in the pan-cancer setting using the Wilcoxon rank-sum test. The prognostic value of GSDM expression in the pan-cancer setting was analyzed using TCGA data. Kaplan-Meier survival curves were constructed to assess whether prognosis was associated with high or low GSDM expression levels, and one-way analysis of variance (ANOVA) was carried out to measure statistical significance. In addition, we further performed a Cox regression analysis to investigate the association between GSDM gene expression and the pan-cancer prognosis data. Finally, we validated the correlation between expression levels and pan-cancer prognosis using data from the Kaplan-Meier plotter database. Based on the TCGA database, we used the receiver operating characteristic (ROC) curve to assess the specificity and sensitivity of GSDM gene expression in cancer, and the areas under the curve (AUCs) were quantified using the “pROC” package of R software. The results were further validated using data obtained from the TARGET (therapeutically applicable research to generate effective treatments) database.

The TME is involved in the occurrence and migration of cancer (Quail and Joyce, 2013). We conducted an analysis of the pan-cancer TME and stemness using the CIBERSORT algorithm and the Spearman’s method, respectively. The immune score and tumor purity analyses were performed using the CIBERSORT algorithm to evaluate the presence of immune infiltrating cells and the tumor purity, respectively, in cancer tissues. Relationship analysis between GSDM family gene expression and the TME and stemness scores was also undertaken using the cor.Test command of the Spearman’s method. We further explored the correlation between GSDM expression and the TMB, and MSI using the Spearman’s correlation test in the selected cancer types. Finally, a drug sensitivity analysis was performed using the “limma” package of R software.

All data analyses and processing were conducted using R software version 4.1.0 (https://www.r-project.org/). The “fdr” algorithm of the “p.adjust” R function has been used to adjust the *p*-value. An adjusted cutoff value of *p* < 0.05 denotes statistically significant differences.

## Results

### Landscape of GSDM gene expression in The Cancer Genome Atlas pan-cancer

**FIGURE 1:**
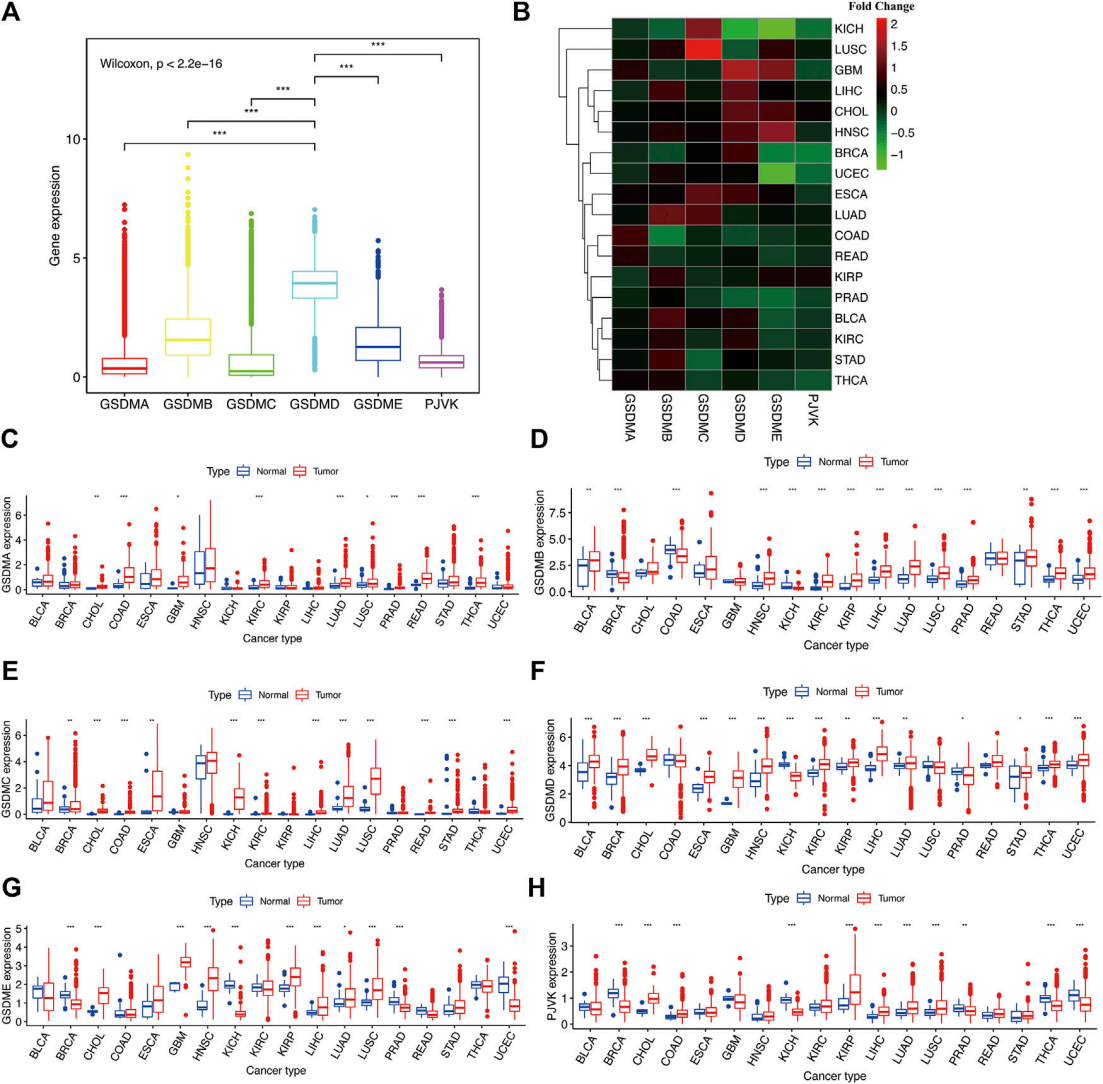
Comparison of the GSDM gene expression landscape in different cancers and corresponding normal tissues. **(A)** Differences in GSDM gene expression in the pan-cancer setting **(B)**. GSDM family gene expression levels in different cancer types (TCGA data); red, high gene expression; green, low gene expression. **(C–H)** Differences in GSDM mRNA levels between normal and cancer tissues. An adjusted cutoff value of *p* < 0.05 denotes statistically significant differences. The asterisks represent statistical significance with adjusted *p* value (*p < 0.05, **p < 0.01, ***p < 0.001). GSDM, gasdermin; TCGA, The Cancer Genome Atlas.

Using the fragments per kilobase of exon model per million reads mapped (FPKM) value of the RNA sequence data from the TCGA project, we explored the mRNA expression levels of GSDM genes in the pan-cancer analysis. According to the results, *GSDMD* exhibited the highest expression levels, followed by *GSDMB*, *GSDME*, and *PJVK*. In contrast, *GSDMA* and *GSDMC* had relatively low expression levels in the tumor specimens (Figure 1A). Subsequently, we utilized the Wilcoxon rank-sum test to compare the mRNA expression levels of GSDM genes in distinct cancer types using both malignant tumor specimen and adjacent normal samples with an adjusted cutoff value of *p* < 0.05 (Figures 1B–H **)** (*p < 0.05, **p < 0.01, ***p < 0.001). We then observed the differences in GSDM mRNA expression levels between tumor/normal sample pairs on a heatmap showing relative fold change in GSDM gene expression (Figure 1B) and boxplots of gene differential expression (Figures 1C–H), for each cancer type. We found that most tumors analyzed and exhibited a certain level of GSDMs expression (Figure 1B); however, the expression of *GSDMD*, *GSDMB*, and *GSDME* in pan-cancers was markedly higher than that of other genes. Interestingly, *GSDMA* and *GSDMC* presented relatively higher gene expression levels in all tumor types compared with matched normal tissues (Figures 1C–E). *GSDMB* expression was significantly lower in tumor specimens of breast invasive carcinoma (BRCA), colonic adenocarcinoma (COAD), and kidney chromophobe (KICH), compared to healthy tissues (Figure 1D). *GSDMD* expression was lower in several cancers, including KICH, lung squamous cell carcinoma (LUSC), and prostate cancer (PRAD), compared to normal tissues (Figure 1F). Relative to normal tissues, *GSDME* expression was higher in most cancer types; however, it was lower in bladder urothelial carcinoma (BLCA), BRCA, COAD, KICH, kidney renal clear cell carcinoma (KIRC), PRAD, thyroid carcinoma (THCA), and uterine corpus endometrial carcinoma (Figure 1G). *PJVK* exhibited lower levels of gene expression in various tumor tissues, except for in cholangiocarcinoma (CHOL), COAD, head and neck squamous cell carcinoma (HNSC), KIRC, kidney renal papillary cell carcinoma (KIRP), liver hepatocellular carcinoma (LIHC), lung adenocarcinoma (LUAD), LUSC, and stomach adenocarcinoma (STAD) (Figure 1H **)**. In summary, GSDM genes showed highly variable gene expression patterns in the pan-cancer setting, indicating that alterations in the expression of GSDM genes may play a crucial role in cancer occurrence and heterogeneity.

### Genetic variation of GSDM genes in The Cancer Genome Atlas pan-cancer samples

**FIGURE 2:**
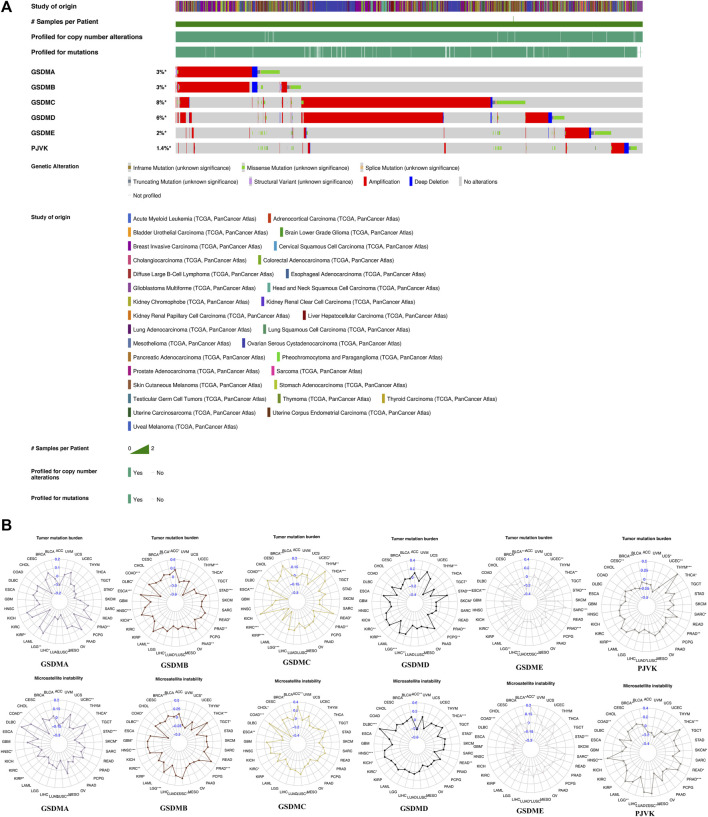
Genetic changes and TME features associated with GSDM genes in the pan-cancer setting. **(A)** Mutation spectrum of the GSDM genes in the pan-cancer setting **(B)** Correlation of GSDM gene expression with TMB and MSI in various cancer types (TCGA data). An adjusted cutoff value of *p* < 0.05 denotes statistically significant differences (*p < 0.05, **p < 0.01, ***p < 0.001). TME, tumor microenvironment; TMB, tumor mutational burden; MSI, microsatellite instability. GSDM, gasdermin.

We next investigated genetic alterations in GSDM genes using TCGA pan-cancer data provided via the cBioPortal database. We found that the overall mutation frequency in GSDM genes was relatively low in pan-cancer tissues, and the most common type of mutation was missense mutations. However, the analysis of copy number variation (CNV) within GSDM genes showed extensive amplification. The genomic landscapes showed that *GSDMC* (8%) and *GSDMD* (6%) had the highest mutational rates in all cancer types, followed by *GSDMA* (3%), *GSDMB* (3%), *GSDME* (2%), and *PJVK* (1.4%) (Figure 2A). Additionally, we focused on the TMB and MSI to examine the potential correlation between the expression of each GSDM gene and various TME features using the Spearman’s correlation test with an adjusted cutoff value of *p* < 0.05 (Figure 2B). Notably, the GSDM gene expression levels were closely related to the TMB in urologic neoplasms (e.g., KIRC, KIRP, and PRAD). A similar significant association between GSDM gene expression and MSI was observed for all cancers examined.

### The association between GSDM gene expression and immune subtypes and clinical traits in pan-cancer

**FIGURE 3:**
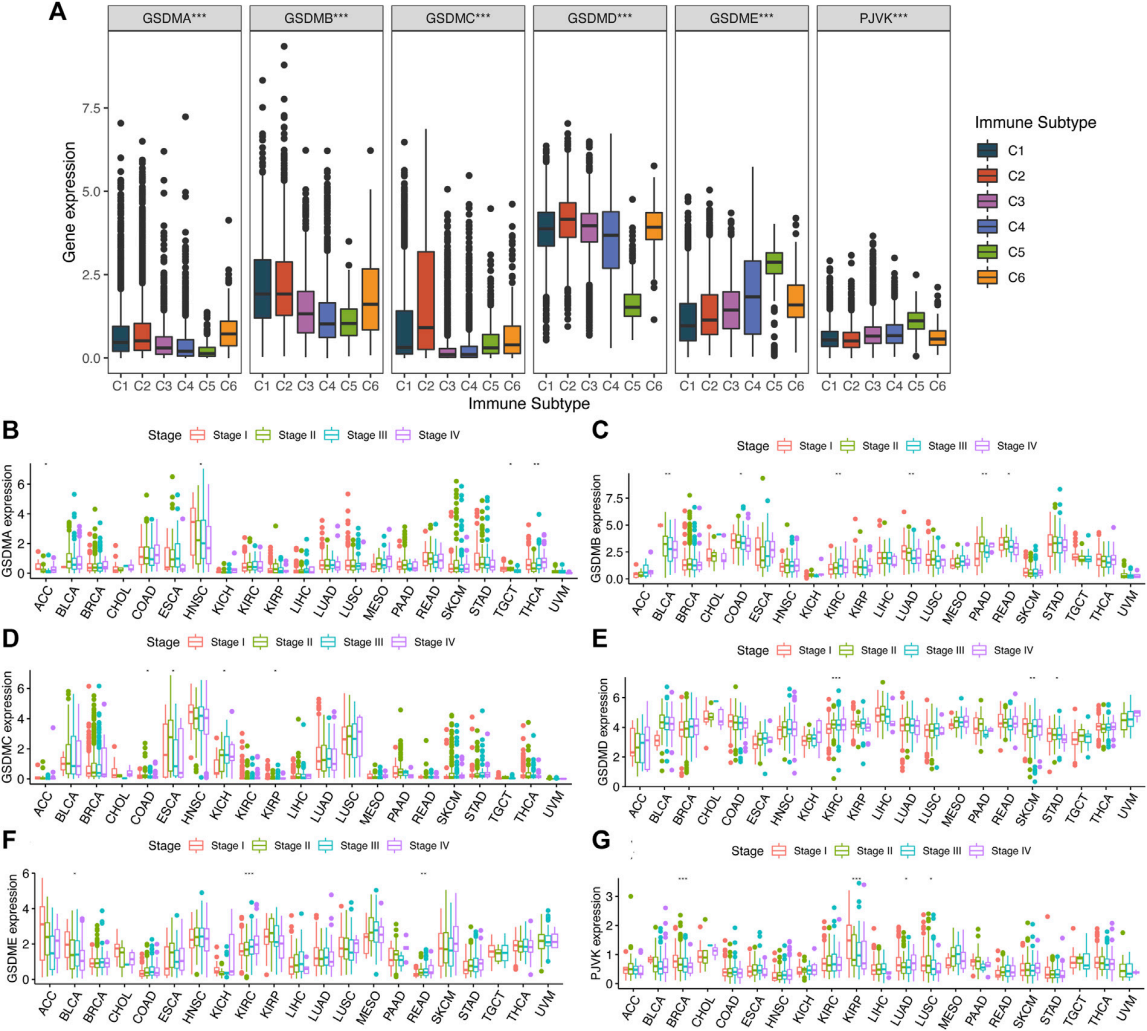
Association between GSDM gene expression and different immune subtypes and clinical traits in the pan-cancer setting. **(A)** Differences in GSDM gene expressions in six pan-cancer immune subtypes: C1 (Wound Healing), C2 (IFN-γ Dominant), C3 (Inflammatory), C4 (Lymphocyte Depleted), C5 (Immunologically Quiet), and C6 (TGF-β Dominant). **(B–G)** Correlation between the tumor stage and GSDM gene expression. An adjusted cutoff value of *p* < 0.05 denotes statistically significant differences (*p < 0.05, **p < 0.01, ***p < 0.001). GSDM, gasdermin; IFN-γ, interferon gamma; TGF-β, transforming growth factor-beta.

To investigate the potential effect of GSDM genes in a pan-cancer setting, we explored the correlation between their mRNA levels and various immune subtypes using TCGA data (Figure 3A). The results demonstrated that the expression levels of GSDM genes differed significantly among the pan-cancer immune subtypes. Genes *GSDMA–D* were more highly expressed in C1 and C2, but less so in C5. The expression of both *GSDMD* and *PJVK* was higher in C4 and C5 (Figure 3A). Next, we performed a clinical correlation analysis of the various cancer types. Overall, the expression of GSDM genes was, as expected, associated with different pathological stages. *GSDMA* showed significant differences in mRNA expression between different clinical stages of four cancers, namely adrenocortical carcinoma (ACC), HNSC, testicular germ cell tumors (TGCT), and THCA (Figure 3B). Relatively high mRNA expression levels of GSDMA were associated with THCA progression; while lower levels were observed in the advanced stages of ACC, HNSC, or TGCT. *GSDMB* mRNA levels differed significantly between the clinical stages of six types of cancer, namely BLCA, COAD, KIRC, LUAD, pancreatic adenocarcinoma (PAAD), and rectum adenocarcinoma (READ) (Figure 3C). Lower *GSDMB* expression tended to be noted in the more advanced stages of BLCA, COAD, LUAD, and READ, while higher *GSDMB* expression was observed in progressive KIRC and PAAD. *GSDMC* mRNA levels varied significantly between the clinical stages of four cancers, including COAD, esophageal carcinoma, KICH, and KIRP (Figure 3D). Higher *GSDMC* expression tended to occur in more advanced stages of KICH. *GSDMD* mRNA levels differed significantly between the clinical stages of KIRC, skin cutaneous melanoma, and STAD (Figure 3E), but were higher in the more advanced stages of KIRC. Furthermore, *GSDME* mRNA levels also varied significantly between different clinical stages of BLCA, KIRC, and READ (Figure 3F), but were consistently higher in the more advanced stages of KIRC and READ. Finally, *PJVK* showed significant differences in mRNA expression at different clinical stages of BRCA, KIRP, LUAD, and LUSC (Figure 3G), while lower *PJVK* expression levels were observed in the more advanced stages of these cancers. The expression levels of GSDM genes were closely associated with tumor progression, involving urinary system cancers in particular. Our findings indicated that the expression levels of GSDM genes were globally associated with the clinical stages of cancer, which has strong implications for the regulation of tumor occurrence and progression to advanced disease.

### Survival analysis in relation to GSDM expression in different cancer types

**FIGURE 4:**
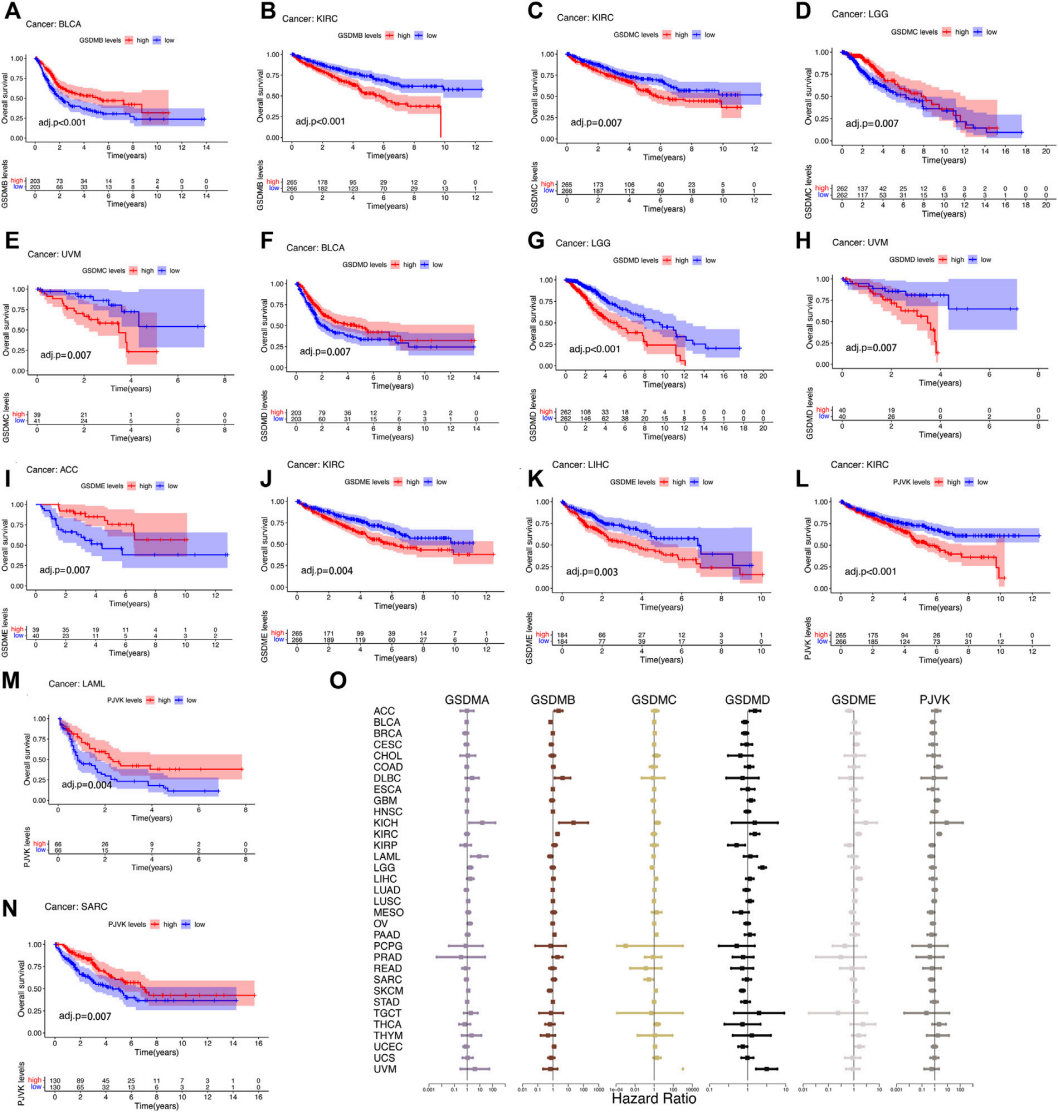
Survival analysis in relation to GSDM gene expression in the pan-cancer setting (TCGA data). **(A–N)** Kaplan-Meier survival curves for the high and low GSDM gene expression groups in the different cancers (TCGA data). **(O)** Cox regression analysis of GSDM gene expression and tumor survival in different cancer types (TCGA data). A hazard ratio <1 and >1 denotes low and high risk, respectively. GSDM, gasdermin; TCGA, The Cancer Genome Atlas.

The prognostic impact of GSDM gene expressions in a pan-cancer setting was analyzed using the Kaplan–Meier survival curve. Using TCGA data, we noticed that the expression of GSDM genes significantly influenced the prognosis of various types of cancer, particularly urinary system cancers (e.g., BLCA, KIRC, and ACC) (Figures 4A–N). Compared to patients exhibiting high GSDM gene expression levels, those with lower gene expression had a marked survival advantage in the majority of cancer types. For example, in patients with KIRC, LIHC, or uveal melanoma, lower GSDM gene expression tended to be associated with better overall survival. However, in BLCA, ACC, acute myeloid leukemia (AML), and sarcoma, higher GSDM expression was linked to a notable survival advantage. In KIRC, lower expression levels of *GSDMB*, *GSDMC*, *GSDME*, and *PJVK* were correlated with better survival. Moreover, we conducted a Cox regression analysis to investigate the value of GSDM genes as prognostic risk factors for each tumor type (Figure 4O). As shown in Table 1, GSDM genes performed as multidimensional factors in cancer prognosis, and their expression significantly predicted the survival of patients with various cancer types, particularly KIRC.

**TABLE 1:** Cox regression analysis of the prognostic values of GSDM genes in the pan-cancer setting.

Gene	Cancer type	HR	95% Confidence interval		*p*-value	Adjusted
GSDMA	KICH	15.58	1.47	165	0.022	0.03
	LAML	9.04	1.98	41.2	0.004	0.012
	LGG	1.74	1.14	2.66	0.011	0.018
	OV	1.58	1.06	2.37	0.026	0.031
	SKCM	1.22	1.06	1.41	0.006	0.014
GSDMB	ACC	2.31	1.26	4.25	0.007	0.014
	BLCA	0.68	0.6	0.78	3.34E−08	4.45E−07
	DLBC	4.21	1.29	13.8	0.018	0.027
	KICH	22.22	2.49	199	0.006	0.014
	KIRC	1.98	1.61	2.42	6.55E−11	1.31E−09
	LAML	0.67	0.48	0.94	0.019	0.027
	PAAD	1.24	1.04	1.48	0.018	0.027
	SKCM	0.62	0.45	0.84	0.002	0.007
GSDMC	KICH	2.25	1.27	3.99	0.005	0.014
	LGG	0.63	0.43	0.93	0.02	0.027
	LIHC	1.56	1.12	2.16	0.008	0.016
	PAAD	1.72	1.32	2.23	6.32E−05	4.21E−04
	SARC	0.28	0.09	0.85	0.024	0.03
	SKCM	1.47	1.18	1.82	5E−04	0.002
	THCA	2.05	1.04	4.05	0.039	0.041
	UVM	1.67E+08	1,068	2.621E+13	0.002	0.007
GSDMD	ACC	1.55	1.12	2.16	0.009	0.017
	KIRC	1.54	1.16	2.03	0.002	0.007
	KIRP	0.51	0.31	0.85	0.01	0.018
	LGG	2.44	1.97	3.02	3.65E−16	1.46E−14
	SKCM	0.72	0.62	0.84	1.72E−05	1.38E−04
	UCEC	0.74	0.56	0.98	0.034	0.038
	UVM	3.15	1.66	5.97	4E−04	0.002
GSDME	ACC	0.67	0.5	0.89	0.007	0.014
	HNSC	1.19	1.02	1.4	0.027	0.031
	KICH	2.89	1.03	8.14	0.043	0.045
	KIRC	1.51	1.21	1.9	3E−04	0.001
	KIRP	0.66	0.45	0.97	0.036	0.039
	LIHC	1.63	1.28	2.08	8.72E−05	4.98E−04
	UCEC	1.64	1.1	2.46	0.016	0.026
PJVK	KIRC	2.31	1.62	3.31	3.69E−06	3.69E−05
	KIRP	0.62	0.4	0.99	0.046	0.046
	LAML	0.54	0.32	0.9	0.019	0.027
	MESO	0.53	0.29	0.95	0.034	0.038
	SARC	0.56	0.34	0.92	0.023	0.03

ACC, adrenocortical carcinoma; BLCA, bladder urothelial carcinoma; DLBC, diffuse large B-cell lymphoma; GSDM, gasdermin; HNSC, head and neck squamous cell carcinoma; HR, hazard ratio; KICH, kidney chromophobe; KIRC, kidney renal clear cell carcinoma; KIRP, kidney renal papillary cell carcinoma; LAML, acute myeloid leukemia; LGG, brain lower grade glioma; LIHC, liver hepatocellular carcinoma; MESO, mesothelioma; OV, ovarian serous cystadenocarcinoma; PAAD, pancreatic adenocarcinoma; PJVK, pejvakin; SARC, sarcoma; SKCM, skin cutaneous melanoma; THCA, thyroid carcinoma; UCEC, uterine corpus endometrial carcinoma; UVM, uveal melanoma.

**FIGURE 5:**
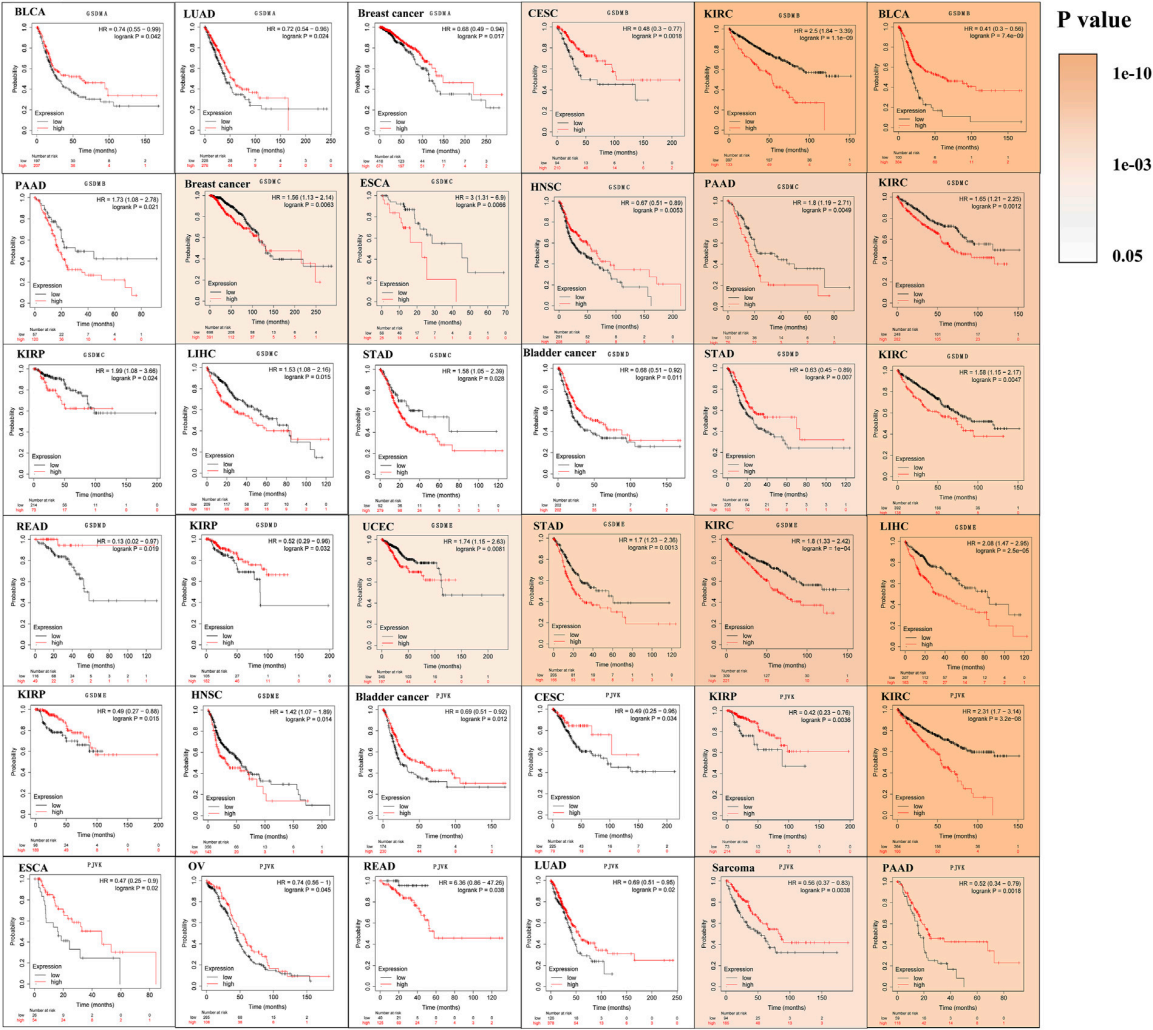
Survival analysis in relation to the expression of GSDM genes in the pan-cancer setting (Kaplan-Meier plotter database). Solid color represents log-rank *p-*value; higher color intensity indicates greater statistical significance. A cutoff value of *p* < 0.05 denotes statistically significant differences. GSDM, gasdermin.

**FIGURE 6:**
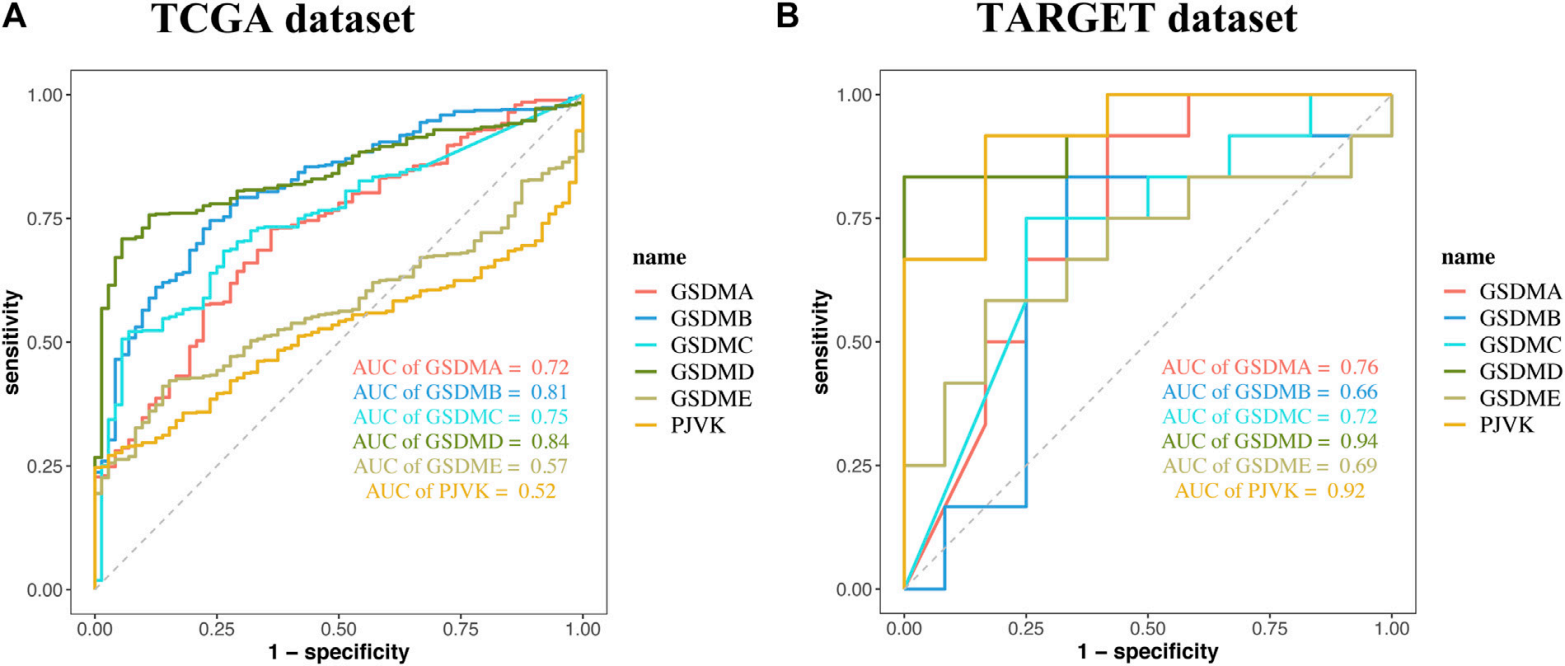
The prognostic performance of GSDM genes. Receiver operating characteristic (ROC) curve showing the prognostic performances of GSDM genes in KIRC using data from TCGA **(A)** and TARGET **(B)** databases. GSDM, gasdermin; KIRC, kidney renal clear cell carcinoma; TARGET, therapeutically applicable research to generate effective treatments; TCGA, The Cancer Genome Atlas.

Analysis of data from the Kaplan-Meier Plotter database revealed that higher *GSDMA* expression was related to better survival in BLCA and breast cancer. The levels of *GSDMB*, *GSDMC*, *GSDMD*, *GSDME*, and *PJVK* were primarily associated with the prognosis of neoplastic disorders of the urinary tract system. Higher *GSEME*, *GSDMD*, and *PJVK* expressions were linked to a survival advantage in KIRP, but were in contrast associated with worse survival in KIRC, STAD, and READ. Interestingly, there was a highly significant correlation between GSDM family gene expression and tumor outcomes in genitourinary cancers, such as KIRC (Figure 5 and Table 2). Subsequently, we assessed the value of GSDM genes in the diagnosis of KIRC. Using TCGA data, we determined that the AUCs of the ROC curves for the prediction of KIRC were 0.72, 0.81, 0.75, 0.84, 0.57, and 0.52 for *GSDMA*, *GSDMB*, *GSDMC*, *GSDMD*, *GSDME*, and *PJVK*, respectively (Figure 6A). Similar results were observed following the analysis of data from the TARGET database (Figure 6B). Collectively, our findings confirm that GSDM genes have significant prognostic value across many different types of cancer, and represent promising biomarkers for cancer therapy.

**TABLE 2:** Significant association between the expression of GSDM genes and the prognosis of different cancer types using data from multiple databases.

Gene	TCGA (cox)	TCGA (Kaplan–Meier)	Kaplan–Meier plotter database
GSDMA	KICH		BLCA
	LAML		LUAD
	LGG		
	OV		
	SKCM		
GSDMB	ACC	KIRC	KIRC
	BLCA	BLCA	BLCA
	DLBC		PAAD
	KICH		CESC
	KIRC		
	LAML		
	PAAD		
	SKCM		
GSDMC	KICH	KIRC	KIRC
	LGG	LGG	KIRP
	LIHC	UVM	ESCA
	PAAD		STAD
	SARC		PAAD
	SKCM		HNSC
	THCA		LIHC
	UVM		
GSDMD	ACC	BLCA	KIRC
	KIRC	LGG	KIRP
	KIRP	UVM	READ
	LGG		STAD
	SKCM		
	UCEC		
	UVM		
GSDME	ACC	ACC	KIRC
	HNSC	KIRC	KIRP
	KICH	LIHC	STAD
	KIRC		LIHC
	KIRP		CESC
	LIHC		HNSC
	UCEC		
PJVK	KIRC	KIRC	KIRC
	KIRP	LAML	KIRP
	LAML	SARC	PAAD
	MESO		ESCA
	SARC		READ

ACC, adrenocortical carcinoma; BLCA, bladder urothelial carcinoma; CESC, cervical squamous cell carcinoma and endocervical adenocarcinoma; DLBC, diffuse large B-cell lymphoma; ESCA, esophageal carcinoma; GSDM, gasdermin; HNSC, head and neck squamous cell carcinoma; HR, hazard ratio; KICH, kidney chromophobe; KIRC, kidney renal clear cell carcinoma; KIRP, kidney renal papillary cell carcinoma; LAML, acute myeloid leukemia; LGG, brain lower grade glioma; LIHC, liver hepatocellular carcinoma; LUAD, lung adenocarcinoma; MESO, mesothelioma; OV, ovarian serous cystadenocarcinoma; PAAD, pancreatic adenocarcinoma; PJVK, pejvakin; READ, rectum adenocarcinoma; SARC, sarcoma; SKCM, skin cutaneous melanoma; STAD, stomach adenocarcinoma; TCGA, the cancer genome atlas; THCA, thyroid carcinoma; UCEC, uterine corpus endometrial carcinoma; UVM, uveal melanoma.

### The association between GSDM gene expression and the TME and stemness score in a pan-cancer setting

**FIGURE 7:**
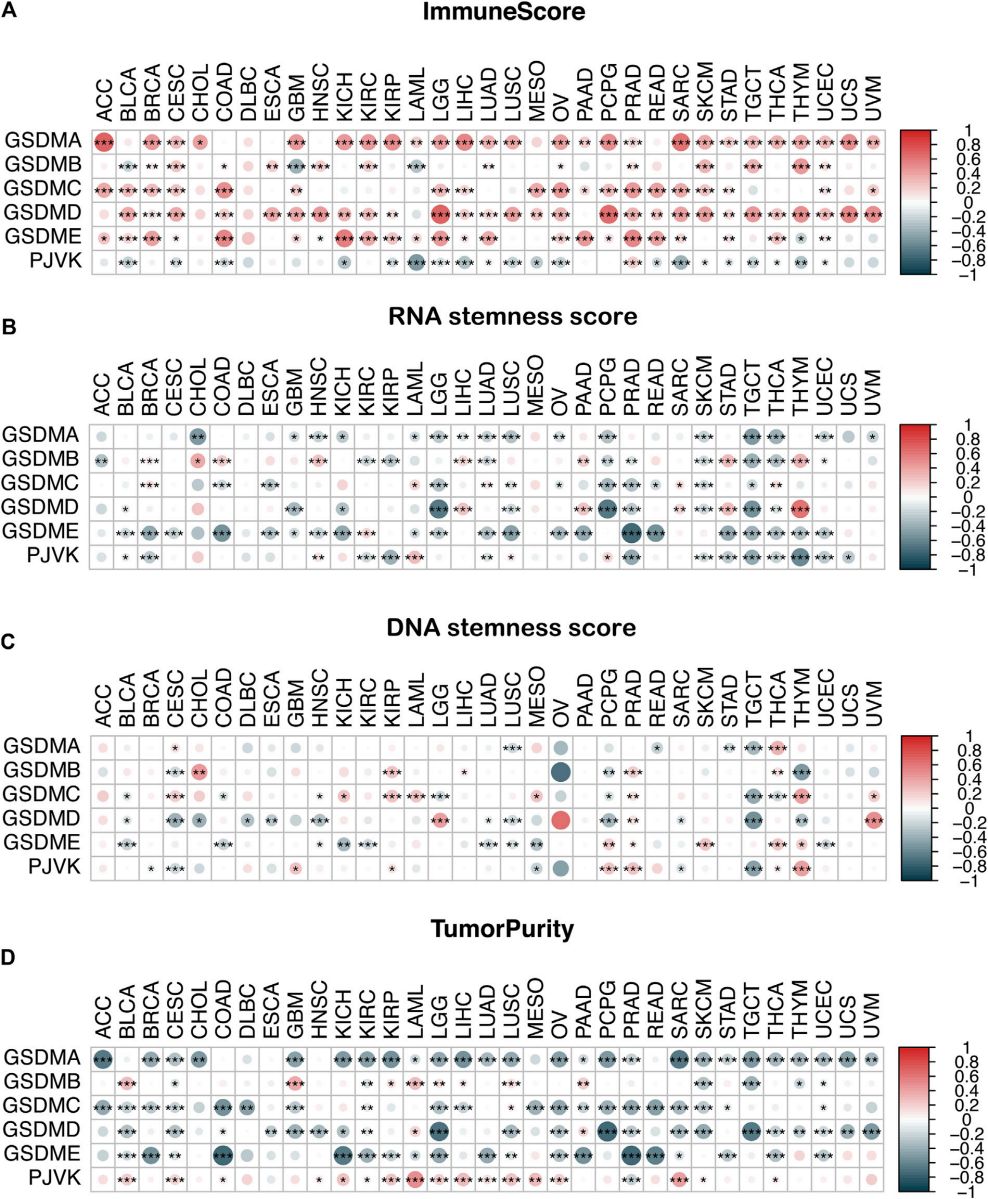
Correlation analysis of GSDM gene expression and the TME and stemness score in the pan-cancer setting. **(A–D)** Association between GSDM gene expression and the immune score **(A)**, the RNA stemness score **(B)**, the DNA stemness score **(C)**, and tumor purity **(D)** in the different cancers; red represents a positive correlation, and blue represents a negative correlation. An adjusted cutoff value of *p* < 0.05 denotes statistically significant differences (*p < 0.05, **p < 0.01, ***p < 0.001). GSDM, gasdermin; TME, tumor microenvironment.

We conducted an immune infiltration analysis and an RNA/DNA stemness score evaluation to explore the correlation between GSDM gene expression and the TME and stemness level in the pan-cancer setting (Figure 7). Our results indicated that the expressions of GSDM family genes, particularly GSDMD, were significantly positively correlated with the immune score (Figure 7A) and negatively correlated with the RNA or DNA stemness score and tumor purity in various cancer types (Figures 7B–D). Notably, *PJVK* expression was negatively correlated with the level of immune infiltration but positively correlated with tumor purity (Figure 7D). These data demonstrate that higher GSDM gene expression (except for *PJVK*) may be associated with an increase in immune cell infiltration into the tumor, together with a decrease in tumor purity and stemness activity, thus potentially improving patient outcomes.

### Association between GSDM gene expression and cancer drug sensitivity

**FIGURE 8:**
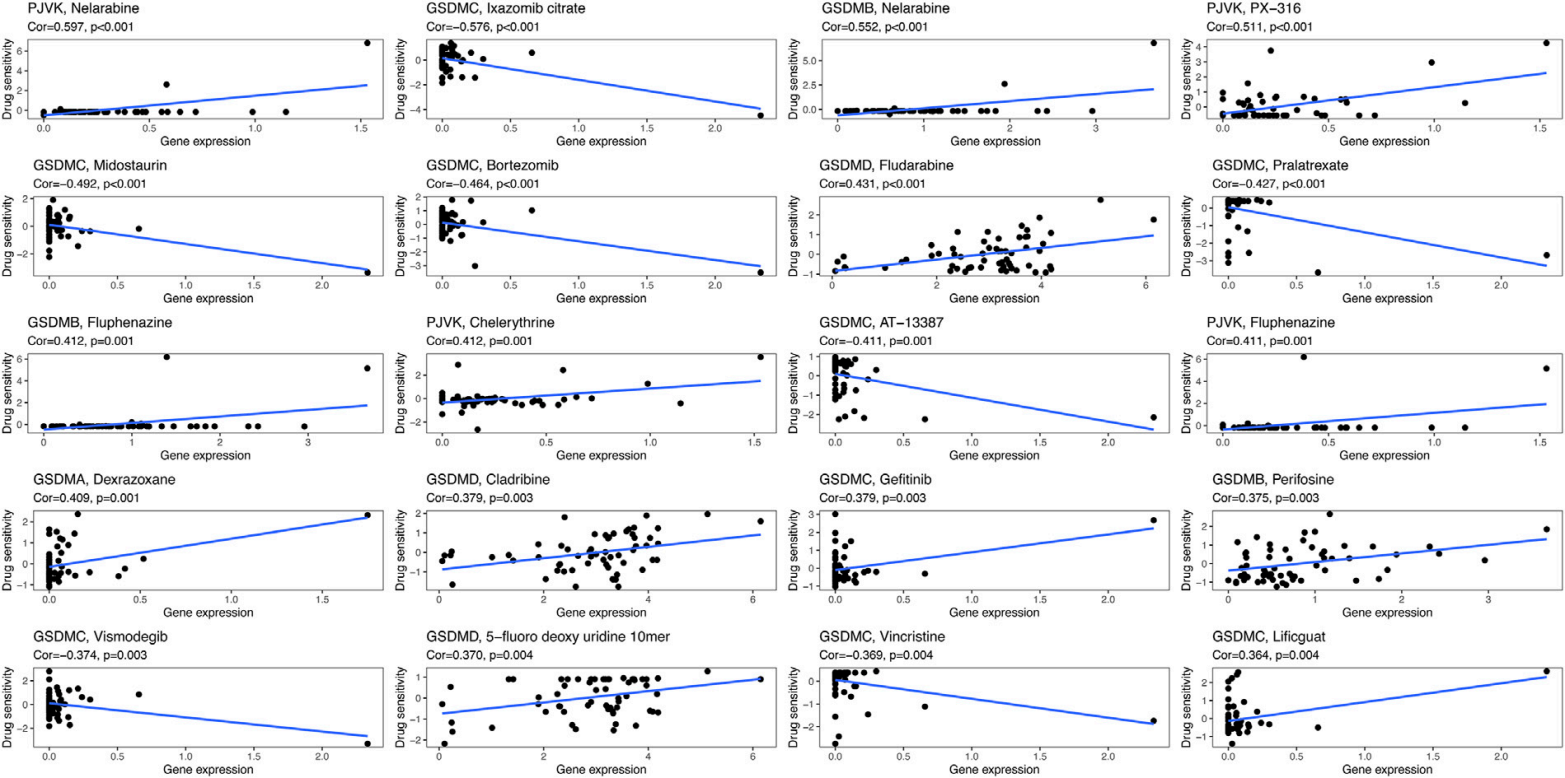
Drug sensitivity analysis of GSDM genes in the pan-cancer setting. GSDM, gasdermin.

We next sought to investigate the potential correlation between the expression of each GSDM gene and the drug sensitivity of cancer cells. For this purpose, we downloaded the drug sensitivity data from the CellMiner™ database, and processed these data using the R software. As illustrated in Figure 8, *GSDMA* expression was highly positively associated with the sensitivity of tumor cells to dexrazoxane, while GSDMB expression showed a markedly positive association with sensitivity to nelarabine, fluphenazine, and perifosine. *GSDMC* expression was negatively correlated with the sensitivity to ixazomib citrate, vincristine, midostaurin, bortezomib, and pralatrexate, as well as positively correlated with sensitivity to gefitinib and lificguat. *GSDMD* expression exhibited a highly positive association with sensitivity to fludarabine and 5-fluoro deoxy uridine 10mer. Finally, *PJVK* expression was positively related to the sensitivity to nelarabine, PX-316, and fluphenazine.

## Discussion

In this study, we conducted a comprehensive analysis of GSDM gene expression across various human cancers and matched normal tissues using multidimensional TCGA data. Based on their membrane pore-forming activity, the GSDM proteins (except PJVK) are important mediators of pyroptosis and participate in tumorigenesis (Tan et al., 2020; Jiang et al., 2021). Recently, it has been reported that two members of the GSDM family, namely *GSDMD* and *GSDME*, stimulate numerous downstream pyroptotic pathways, and the downregulation of these molecules conversely contributed to tumorigenesis and proliferation in the tumor cell microenvironment (Wang et al., 2018a; Gao et al., 2018; De Schutter et al., 2021). Although previous studies have investigated the gene expression patterns of some pyroptosis genes in tumors and normal tissues (Hu et al., 2021; Qiu et al., 2021), their analyses were restricted to a specific cancer type. Therefore, the objectives of the present analysis were to determine the expression patterns of GSDM genes in numerous cancer types and adjacent normal tissues, as well as evaluate their association with immune infiltration, genetic variation, and drug sensitivity. We hope that our work will provide new insights into the use of GSDM genes as pan-cancer biomarkers.

Programmed cell death plays pivotal role in coordinating homeostasis and cell proliferation in malignant disorders, such as tumors and inflammatory or infectious diseases (Bedoui et al., 2020). Pyroptosis is a form of programmed cell death that triggers lytic cell rupture in order to maintain homeostasis via an intricate cascade of potassium efflux, water influx, and cellular swelling processes, all of which are dependent on GSDM family proteins (Bedoui et al., 2020; Broz et al., 2020). Six GSDM family genes (i.e., *GSDMA*, *GSDMB*, *GSDMC*, *GSDMD*, *GSDME*, and *PJVK*) have long been described in other fields; however, they have been rarely investigated in a pan-cancer setting. Thus far, it has been reported that GSDMA is involved in skin inflammation (Zhou et al., 2012), epidermal differentiation (Lin et al., 2015), and the development of asthma (Ferreira et al., 2014). Moreover, low expression levels of the GSDMA protein have been found in gastric cancers (Saeki et al., 2000). Upregulation of *GSDMB* participates in the pathogenesis of breast, hepatic, cervical, and gastrointestinal cancers (Sun et al., 2008; Hergueta-Redondo et al., 2014) and has been linked to poor patient survival. *GSDMC* and *GSDMD* are markedly upregulated in breast cancer and colorectal cancers (Miguchi, et al., 2016; Hou et al., 2020), where they promote tumor cell proliferation and are associated with a worse prognosis (Wang et al., 2019). Elevated *GSDME* activity exerts suppressive effects on carcinogenesis and cancer progression (Wang et al., 2018b). Our analysis of TGGA data demonstrated that GSDM genes (*GSDMB*, *GSDMC*, and *GSDMD* in particular) were upregulated in different types of cancers compared with the corresponding normal specimens. Furthermore, a marked increase in GSDM gene expression was observed in the majority of kidney carcinomas that progressed from early to advanced clinical stages.

An unrestrained inflammatory response is highly amplified in the TME, and the excessive activation of cytokine secretion pathways leads to pathological consequences. The release of pro-inflammatory cytokines is crucially dependent on the activation of GSDM genes (Heilig et al., 2018; Li et al., 2021; Liu et al., 2021). GSDM-meditated pyroptosis culminates in the release of tissue factor, which activates tumor inflammation pathways, leading to poor outcomes in patients with cancer. In fact, the GSDM genes are also closely associated with tumor immunity. For example, the tumor-suppressive effect of *GSDME* is mediated by tumor-infiltrating natural killer (NK) and CD8+T lymphocytes (Zhang et al., 2020). Meanwhile, the overexpression of *GSDMB* in cancer cells leads to the recruitment of immune cells and promotes tumor mobility and invasion (Hergueta-Redondo et al., 2014; Cui et al., 2021). We have observed that the expression of GSDM genes is amplified in many human cancers, particularly kidney cancer. Heightened GSDM gene expression may lead to the activation of different stimuli and inflammatory caspases, and trigger the infiltration of immune cells, thus reflecting the complex role of pyroptosis in tumorigenesis, antitumor immunity, tumor cell growth, and metastasis. Therefore, our research suggests that GSDM gene family members may function as oncogenes in the pan-cancer setting and could represent potential prognostic biomarkers and immunotherapeutic targets in the treatment of kidney cancer in particular.

We next explored the genetic alterations affecting GSDM genes in various types of cancer. In general, a higher frequency of somatic mutations is thought to be associated with the increased generation of more neoantigens. The TMB is defined as the number of coding, and somatic mutations per megabase of the interrogated genome. It is similar to high-MSI in cancer, and is involved in the generation of immunogenic neuropeptides on the tumor cell surface, thus influencing patient responses to immunotherapy (Yamamoto and Imai, 2019; Huang et al., 2021). Currently, TMB and MSI are predictors of the efficacy of immunotherapy. In this study, we found that the mutational frequencies within GSDM genes were at an extremely low level, and that the expression levels of GSDM genes were significantly correlated with TMB and MSI across various urologic neoplasms. This result suggests that the GSDM genes may be stable biomarkers and should be considered in potential treatment strategies for cancer.

Subsequently, we performed survival and Cox regression analyses, which revealed that the GSDM genes were significantly associated with the prognosis of urinary tract system cancer (e.g., KIRC, PAAD, KIRP, and BLCA). For instance, downregulated GSDM genes were related to favorable survival outcomes in KIRC, while some of the upregulated GSDM genes predicted better KIRP and BLCA prognoses. In addition, we found that the expression levels of GSDM genes were more frequently correlated with the prognosis of KIRC. We subsequently analyzed the predictive value of the GSDM genes in KIRC. The results showed that *GSDMD* presented the highest AUC score, followed by *GSDMB* and *GSDMC*. These findings indicated that the diagnostic value of GSDMD may be better than that of the other GSDM genes. KIRC is a highly infiltrative tumor that remains one of the most sensitive tumors in terms of response to immunotherapy (Inman et al., 2013; Newman et al., 2015). It has been reported that some spontaneous KIRC regressions are accompanied by signs of immune mediation (Janiszewska et al., 2013). The GSDM genes are involved in cell programmed cell death; and *GSDMD* in particular, plays an important role in systemic immune-inflammatory sensing (Yao et al., 2022). Dysregulation of the GSDM genes may cause a dysfunctional adaptive immune response, as well as contribute to both the initiation and progression of multiple tumors (Xia et al., 2019; Fang et al., 2020). Previous studies have shown that the upregulation of *GSDMD* in tumor tissues was associated with a poor prognosis, due to its involvement in AKT-related signaling pathways (Wang et al., 2018a; Gao et al., 2018). Thus, we hypothesized that *GSDMD* may trigger the release of inflammatory factors and induce potential interactions with other immune responses, thereby promoting the invasiveness of kidney tumors. These findings imply that the dysregulations of GSDM genes could predict survival in patients with cancers, and that *GSDMD* may represent a robust biomarker for the evaluation of KIRC.

We then went on to conduct a potential correlation analysis between the expression of the GSDM gene and the immune infiltration, immune subtype, or RNA/DNA stemness score in the pan-cancer setting. Previously, Thorsson et al. performed a comprehensive immunogenomic analysis of all TCGA cancer types and successfully identified six immune subtypes (C1–C6) (Thorsson et al., 2018). Through our research, we discovered that higher expression levels of *GSDMA*, *GSDMB*, *GSDMC*, and *GSDMD* were more closely related to hyperimmune subtypes, such as C1, C2, and C6. Lower GSDM expression levels (except for *PJVK* and *GSDME*) were correlated with hypoimmune subtypes, such as C4 and C5. Consistent with these findings, the immune infiltration analysis showed that GSDM gene expressions were significantly positively correlated with the immune cell infiltration score of nearly all cancer types. Thus, GSDM-dependent pyroptosis could increase immune cell activation within the tumor, thereby contributing to immune-mediated tumor cell regression (Tsuchiya, 2021). Moreover, recent studies have confirmed that the stemness index (RNA/DNA stemness score) was significantly higher in patients with metastases and disease recurrence. The stemness score was also correlated with intratumor heterogeneity, immune response, and drug resistance (Malta et al., 2018; Yi et al., 2020; Shi et al., 2021). We used two independent stemness indices, namely the DNA stemness score (reflecting epigenetic features) and RNA stemness score (reflecting mRNA gene expression) (Malta et al., 2018). Previous reports have found that lower stemness indices were correlated with an increased leukocyte fraction and higher programmed cell death-ligand 1 (PD-L1) expression levels (Zaretsky et al., 2016; Malta et al., 2018). In this study, we observed a more negative correlation between the tumor stemness and GSDM gene expression in most cancer types. We argue that such tumors would be more susceptible to immune checkpoint blockade due to sufficient immune infiltration and the up-regulation of the PD-L1-associated gene pathway, which further enhances treatment efficacy (Zaretsky et al., 2016). However, the survival analysis indicated that patients with kidney cancer (e.g., KIRC) and a higher level of GSDM gene expression were associated with a significantly poorer prognosis. These findings may be explained by the immune-excluded phenotype of the TME, which is characterized by an abundance of immune cells in the TME that are retained in the stroma and do not penetrate tumor cell nests. Therefore, immune cell infiltration appeared to occur outside the tumor (Salmon et al., 2012; Joyce and Fearon, 2015). Based on these results, we hypothesized that the evaluation of the stemness index and the immune infiltration score of GSDM-mediated cancer pyroptosis may provide more effective immunotherapeutic options for cancer.

Finally, we analyzed the correlation between the expression of GSDM genes and drug sensitivity in 33 cancer types using data from the CellMinerTM database. The results showed that the expression of GSDM genes positively correlated with the sensitivity of tumor cells to nelarabine, fluphenazine, and dexrazoxane. However, higher expression levels of these genes were also linked to reduced cell sensitivity to bortezomib, midostaurin, and vincristine. Nelarabine is an effective anticancer chemotherapy prodrug of arabinfuranosylguanine triphosphate, that appears to meditate DNA degradation and cell death (Robak et al., 2006). Similarly, midostaurin (Hsiao et al., 2019), fluphenazine (Otręba and Kośmider, 2021), bortezomib (Vora et al., 2020), and vincristine (Xu and Xu, 2020) also possess anticancer activity. The present findings revealed that GSDM gene expression may provide important guidance for the selection of targeted drugs for the treatment of cancer.

This study has some limitations. Our analyses were based on the mRNA expression level data extracted from online databases, and the results were obtained using bioinformatic methods. Therefore, we lacked evidence from *in vitro* or *in vivo* experiments to support these findings. Further investigation is warranted to validate our findings and to elucidate the roles of GSDM genes in cancer.

In summary, our work revealed statistically significant variations in GSDM mRNA expression levels between different tumor tissues and healthy human organs. We also observed that the GSDM genes participated in tumorigenesis, as they were correlated with tumor immune subtypes, patient survival, the TME, and the stemness score. Our pan-cancer analysis indicates that the expression of GSDM genes (in particular *GSDMB*, *GSDMC*, and *GSDMD*) was significantly associated with the survival of patients with certain types of urinary tract system cancer. Thus, GSDM genes may represent potential prognostic biomarkers for these cancers. Of the six GSDM family members, GSDMD, in particular, has shown promise as a biomarker for the evaluation of KIRC. Moreover, we found that the expression levels of GSDM genes in tumor cell lines were correlated with varying sensitivities of specific cancer drugs. These findings may provide new insights into the potential use of GSDM genes as therapeutic targets in the pan-cancers setting.

## Data Availability

Publicly available datasets were analyzed in this study. This data can be found here: https://xena.ucsc.edu/, http://www.cbioportal.org, https://discover.nci.nih.gov/cellminer/home.do, https://gtexportal.org/home/, and https://www.cbioportal.org.
